# Uncovering natural variation in *TdBCAT* genes and phenotypic variation under drought in durum wheat

**DOI:** 10.3389/fpls.2026.1826616

**Published:** 2026-09-14

**Authors:** Valentina Buffagni, Donatella Danzi, Ricardo Humberto Ramirez-Gonzalez, Cristobal Uauy, Mariolina Gullì, James Simmonds, Angelo Petrozza, Francesco Cellini, Stephan Summerer, Domenico Pignone, Michela Janni, Nelson Marmiroli

**Affiliations:** 1 Department of Chemistry, Life Sciences and Environmental Sustainability, University of Parma, Parma, Italy; 2 John Innes Centre, Norwich, United Kingdom; 3 Institute of Biosciences and Bioresources (IBBR)-Consiglio Nazionale delle Ricerche (CNR), Bari, Italy; 4 ALSIA Centro Ricerche Metapontum Agrobios, Metaponto, MT, Italy; 5 Institute of Materials for Electronics and Magnetism IMEM-Consiglio Nazionale delle Ricerche (CNR), Parma, Italy; 6 CINSA, Interuniversity Consortium for Environmental Sciences, Parma, Italy

**Keywords:** drought, durum wheat germplasm, genotype selection, genotyping, landraces, phenotype selection, phenotyping

## Abstract

Drought severely limits wheat productivity, highlighting the need for tolerant genetic resources. Targeted allele mining and high-throughput phenotyping (HTP) were combined to assess natural variation in *TdBCAT* genes and drought response in 38 durum wheat genotypes. A cost-effective strategy integrating next-generation sequencing and Kompetitive Allele Specific PCR (KASP) genotyping identified 27 single nucleotide polymorphisms (SNPs) and 10 haplotypes across *TdBCAT-A* and *TdBCAT-B*, mainly in promoter regions. Under controlled drought, RGB imaging and the digital biovolume ratio (DBR) classified SSD 195, SSD 269, SSD 322, SSD 343, SSD 409, SSD 416, SSD 441, and Svevo as the best-performing genotypes, with SSD 195 and SSD 441 showing the greatest stability. Integrating genotypic and phenotypic data identified *TdBCAT* haplotype combinations associated with improved drought performance and valuable landraces for pre-breeding. This integrated approach efficiently characterized haplotypic diversity and identified drought-adaptive germplasm, providing valuable resources for pre-breeding and the sustainable selection of durum wheat under water-limited conditions.

## Graphical Abstract

**Figure:**
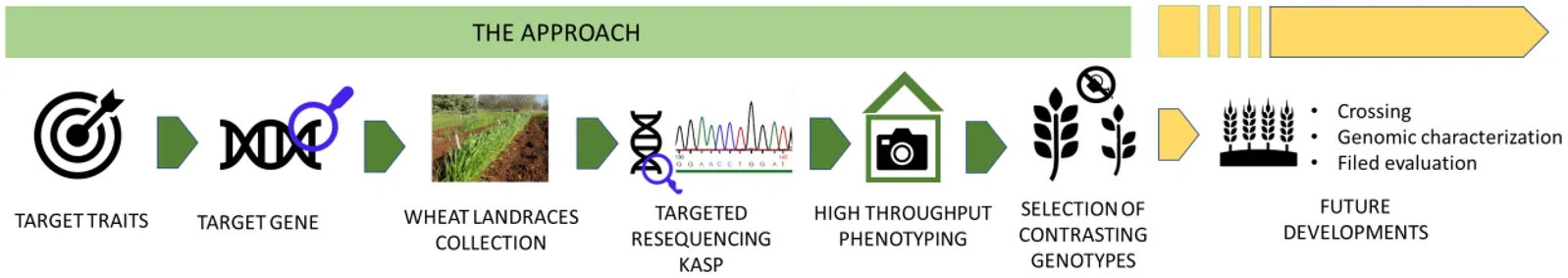
Combining a novel genomics approach with phenotyping analysis to identify natural variation within the *TdBCAT* sequence in a panel of durum wheat genetic resources (SSD lines).

## Introduction

Drought stress (DS) represents a major challenge in the context of global climate change, significantly affecting crop production, particularly in arid and semi-arid regions (Raza et al., 2019; Zenda et al., 2022). With the global population projected to double by mid-century, the need to increase wheat production becomes increasingly urgent (United Nations, Department of Economic and Social Affairs, Population Division, 2024; Wheat Initiative, 2023), requiring the identification of new wheat varieties capable of adapting to changing water availability.

Durum wheat (*Triticum turgidum* L. ssp. *durum*), a staple crop domesticated over 8,000 years ago, has been widely cultivated across North Africa, South Europe, and West Asia due to its adaptability to diverse environments, long shelf life, and versatility in food processing (Curtis, 2002; Sansaloni et al., 2020). However, this essential crop is highly vulnerable to drought, with severe impacts on yield and grain quality (Helman and Bonfil, 2022; Wan et al., 2022; Sareen et al., 2023). Mitigating drought effects requires both the improvement of water-use efficiency at the whole-plant level (Ismael et al., 2022) and the exploitation of genetic diversity from germplasm collections to introgress elite cultivars with adaptive traits (Maccaferri et al., 2011, 2019; Condorelli et al., 2018; Bassi and Nachit, 2019; Allier et al., 2020; He and Li, 2020; Khadka et al., 2020; Morgounov et al., 2021). Although wheat breeding has historically focused on yield-related traits, this strategy has often reduced environmental resilience (Pignone and Hammer, 2013), highlighting the current need to restore balance between productivity and stress adaptation (Abidi et al., 2024).

Globally, *ex situ* wheat germplasm collections maintained in national and international gene banks represent a vast reservoir of natural variation (Cheng et al., 2024). Integrating passport, climatic, and phenotypic data enables the identification of genotypes harboring adaptive traits for drought tolerance. In this framework, international initiatives such as the Wheat Initiative and the Global Durum Wheat Panel have provided representative collections capturing up to 94%–97% of the original genetic diversity in durum wheat (Mazzucotelli et al., 2020), offering unprecedented opportunities for pre-breeding and genetic improvement.

The selection of drought-tolerant genotypes remains inherently slow and complex. However, the integration of high-throughput sequencing and high-throughput phenotyping (HTP) has dramatically accelerated the dissection of genotype–phenotype relationships under stress (Bhat et al., 2016, 2020; Varshney et al., 2018, 2021; Janni and Pieruschka, 2022). Recent advances in digital phenotyping and image-based trait extraction have further improved the precision of DS quantification, enabling the dynamic monitoring of plant growth, biomass retention, and water status under controlled and field conditions (Palermo et al., 2024, 2026). These approaches provide robust phenotypic proxies for drought resilience and support the identification of adaptive traits at early developmental stages.

In parallel, allele mining has emerged as an effective strategy to identify novel functional variants within candidate genes relevant to stress tolerance (Janni et al., 2020; Sinha et al., 2020). The integration of omics technologies, including genomics, transcriptomics, metabolomics, and phenomics, enables the association of genetic variation with drought-responsive phenotypes (Wang et al., 2013, 2021; Kuzmanović et al., 2014, 2020; Kabbaj et al., 2017; Sallam et al., 2019; Beena et al., 2021; Shen et al., 2021).

This study focuses on the *TdBCAT* gene family, previously identified as a key component of drought response in durum wheat (Buffagni et al., 2020). Transcriptomic and metabolic analyses have demonstrated cultivar-specific regulation of *BCAT* genes under water deficit, suggesting their involvement in adaptive mechanisms (Razzaq et al., 2022). By combining targeted next-generation sequencing (NGS) with high-throughput phenomics, we investigated natural allelic variation at the *TdBCAT* loci in a core set of durum wheat germplasm and evaluated its association with drought-related phenotypes. This integrated approach aims to identify adaptive haplotypes and elite landraces that can be exploited for the development of drought-resilient wheat varieties under future climate scenarios.

## Materials and methods

### Plant growth and treatment conditions

A total of 33 *Triticum durum* genotypes were selected out of 452 genotypes from the single seed descent (SSD) collection held by IBBR-CNR in Bari (Pignone et al., 2015). Five commercial varieties (Svevo, Saragolla, Cappelli, Colosseo, and Kronos) were also included for the allele mining approach for a total of 38 genotypes.

The SSD genotypes used in this study are part of the Mediterranean Germplasm Database (MGD) maintained by the Institute of Biosciences and Bioresources (IBBR, CNR) and are listed in **Supplementary Table 1**. For each genotype, the corresponding MGD accession number is provided, enabling retrieval of the associated passport data from the MGD. The country of origin of each genotype is also reported in **Supplementary Table 1**.

From the original collection of 452 SSD genotypes established in 2015 and described by Pignone et al. (2015) a core collection of 32 SSD genotypes was subsequently selected based on phenotypic data (Danzi et al., 2019) and further characterized by Palermo et al. (2024). Passport information for all accessions is available through the Mediterranean Germplasm Database (MGD; https://www.ibbr.cnr.it/mgd/?action=search&p=str).

The germplasm analyzed in the present study comprised a subset of the original core collection, selected according to seed availability. Genomic analyses were performed on 33 SSD genotypes together with five reference cultivars (Kronos, Colosseo, Cappelli, Saragolla, and Svevo) for a total of 38 genotypes.

Phenotypic analyses were conducted on 32 SSD genotypes, as SSD 499 was excluded owing to the lack of seed material, and on three reference cultivars (Cappelli, Saragolla, and Svevo) for a total of 35 genotypes.

To retrieve allele variation within the *TdBCAT* sequence, plants were grown with regular irrigation to field capacity, and DNA was extracted in the following section.

For the HTP analysis, the description of the plant growth conditions and treatment is listed in the dedicated section.

### Genomic DNA and gene targeting

Genomic DNA was extracted from leaves of durum wheat with the GenElute™ Plant Genomic DNA Miniprep Kit (Sigma-Aldrich). DNA samples were quantified and adjusted to similar concentrations by running genomic DNA on agarose gel with lambda DNA standards of known concentrations.

A targeted resequencing approach was applied to sequence a panel of genes of interest using NGS before identifying the full-length target sequences (**Figure 1**) (Buffagni et al., 2020). This approach identifies multiple overlapping PCR products covering the entire target sequences of both *TdBCAT-A* and *TdBCAT-B* genes (**Supplementary Figure 1**). The combination of targeted NGS and Kompetitive Allele Specific PCR (KASP) markers (Buffagni et al., 2020) leads to a simple and cost-effective allele mining strategy. Samples were pooled to reduce NGS costs, and KASP markers facilitated single nucleotide polymorphism (SNP) detection within target sequences, ensuring accurate allele assignment to specific genotypes. This study focused on 38 selected genotypes, 32 of which belong to an SSD collection of durum wheat landraces (Pignone et al., 2015; Danzi et al., 2019). These genotypes were selected based on prior phenotypic analysis of the core SSD collection (131 genotypes) for different drought tolerance behaviors (Danzi et al., 2022).

**Figure 1:**
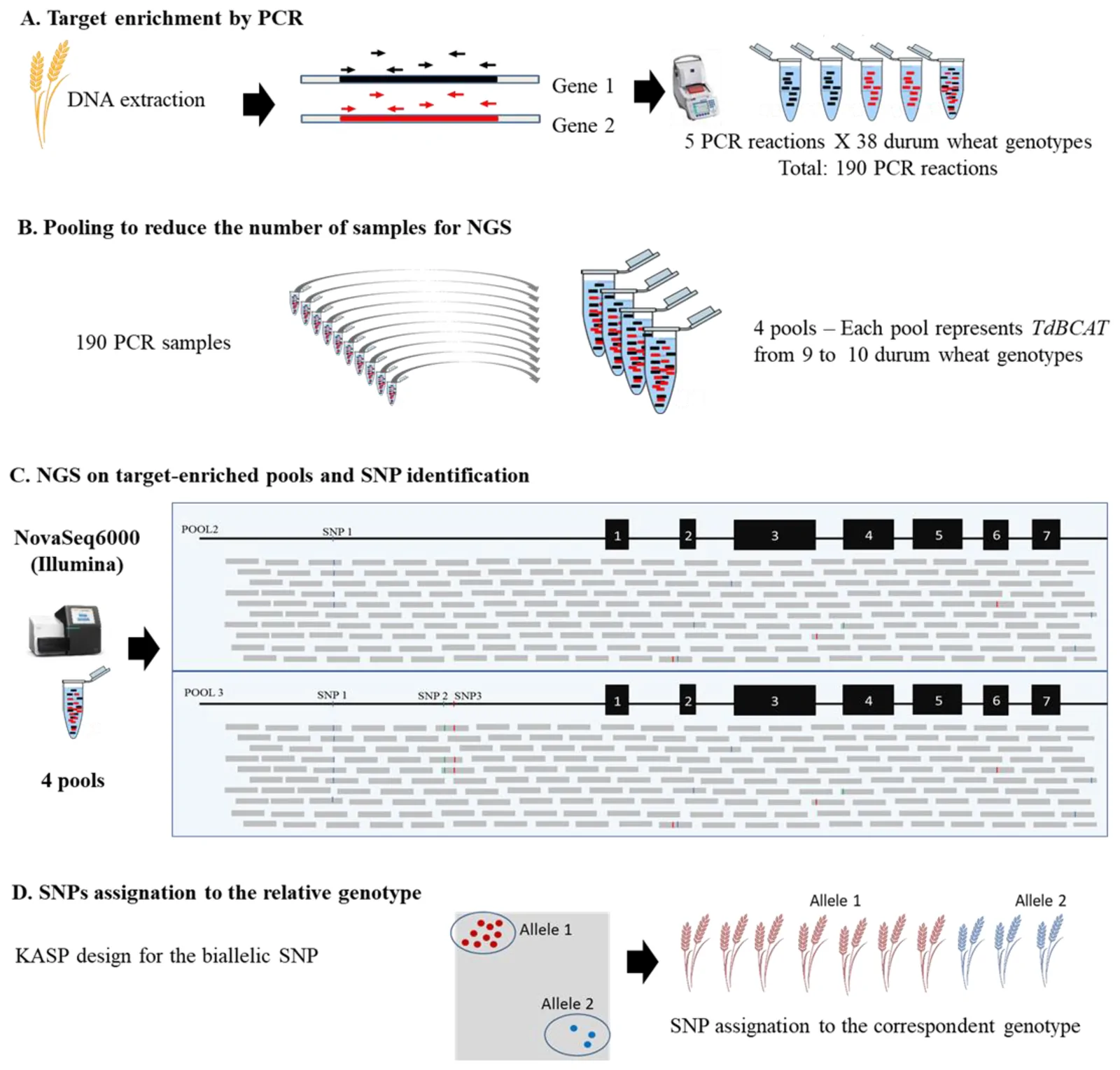
Scheme of the method used for the allele mining. **(A)** Targeted enrichment by PCR is followed by **(B)** a two-step pooling to reduce the number of samples for the **(C)** sequencing by NGS. After the alignments of all the reads to the reference sequence, the identified SNPs are finally **(D)** assigned to the corresponding genotype with a KASP assay.

*TdBCAT-A* and *TdBCAT-B* genes were chosen for their role in drought tolerance in durum wheat (Buffagni et al., 2020).

Five PCRs were performed to target *TdBCAT-A* and *TdBCAT-B* sequences on 33 genotypes of durum wheat landraces along with five commercial varieties (Svevo, Saragolla, Cappelli, Kronos, and Colosseo) according to the scheme in **Supplementary Figure 1** (Buffagni et al., 2020). Briefly, primer design included five primer pairs (PPs) targeting proximal promoter regions and complete coding sequences (CDSs) of both homeologs of *TdBCAT* (total 6.8 Mb). PPs were designed to either be homeolog-specific (from PP1 to PP4) or target both A and B homeologs of *TdBCAT* in one reaction (PP5). The list of PPs and optimized annealing temperatures is reported in **Supplementary Table 2**.

PCR was performed in 25 μL final volume, using TaqDNA Polymerase (New England BioLabs, Hitchin, UK), 10–20 ng of template DNA, 0.2 μM of each forward and reverse primers, 0.2 mM of each dNTP, and 1× Standard Taq Buffer (New England BioLabs).

PCR amplifications with PP1, PP2, and PP5 were performed with a touch-down PCR as follows: (1) initial denaturation at 95°for 2 min; (2) 10 cycles at 95 °C for 30 s, annealing at 65 °C for 30 s with a drop −0.5 °C/cycle, and elongation at 72°C for 2 min; (3) 35 cycles of 94 °C for 30 s, annealing for 30 s, and elongation at 72 °C for 2 min; and (4) one cycle at 72 °C for 10 min.

PCR amplifications with PP3 and PP4 were performed as follows: (1) initial denaturation at 95 °C for 2 min; (2) 35 cycles at 95 °C for 30 s, annealing for 60 s, and elongation at 72 °C for 2 min; and (3) 1 cycle at 72 °C for 10 min.

Amplification of expected PCR products was confirmed by agarose gel electrophoresis and confirmed by sequencing with the BigDye™ Terminator v3.1 Cycle Sequencing Kit (Thermo Fisher Scientific, Waltham, MA). PCR products amplified by PP5 were digested with *Bmg*BI (New England BioLabs) and checked on agarose gel to assess the presence of both targets from A and B genes.

### Pooling DNA for target sequencing

Each PCR product was quantified by Nanodrop 8000 (Thermo Fisher Scientific) and adjusted to similar concentration across different samples. PCR products were then pooled together following a two-step procedure. During the first step, PCR amplicons from the same DNA were pooled into one tube, for a total of 38 pools (including 33 SSDs and five varieties). After a concentration adjustment, a second step of pooling was performed to reduce the final number of samples for NGS analysis. Thus, four pools were obtained to include complete target sequences from 38 genotypes as follows: pool 1 (SSD 35, SSD 92, SSD 122, SSD 171, SSD 253, SSD 269, SSD 416, SSD 487. SSD 494, and cv. Kronos); pool 2 (SSD 44, SSD 109, SSD 178, SSD 322, SSD 343, SSD 397, SSD 415, SSD 511, and cv. Colosseo); pool 3 (SSD 69, SSD 99, SSD 116, SSD 244, SSD 335, SSD 409, SSD 441, SSD 451, cv. Cappelli, and cv. Saragolla); and pool 4 (SSD 64, SSD 112, SSD 135, SSD 195, SSD 278, SSD 325, SSD 459, SSD 499, and Svevo).

### Sequencing data analysis and haplotype reconstruction and assignment

These last four pools were sequenced with Illumina NovaSeq6000 (Novogene, Beijing, China). The paired end (PE) reads raw data were checked for quality control (QC) by using FastQC (http://www.bioinformatics.babraham.ac.uk/projects/fastqc/). Fastq files were aligned to Kronos reference genome v.1.1 (https://opendata.earlham.ac.uk/opendata/data/Triticum_turgidum/EI/) using BWA default settings (version 0.7.17) to generate Sequence Alignment Map files (SAM). Samtools (version 1.7) was used to convert SAM to sorted BAM (the binary version of SAM) files for each sample. BAM files were directly visualized with the Integrative Genomic Viewer IGV v. 2.4 (Broad Institute) for SNP identification and selection. SNPs identified from pooled targeted resequencing were used to reconstruct haplotypes for *TdBCAT-A* and *TdBCAT-B*. Each sequencing library represented PCR amplicons derived from 9–10 highly homozygous SSD genotypes. Following alignment to the Kronos reference genome, polymorphic sites were identified using IGV v2.4. Because DNA contributions were balanced during the two-step pooling procedure, the frequency of variant reads was used as an indicator of the number of genotypes carrying a given SNP within each pool. For example, an SNP present in a single genotype within a pool of 10 genotypes was expected to occur at approximately 10% of allele frequency. Any SNP having <5% of different nucleotides between called reads and the reference genome were considered unreliable and thus eliminated from the candidate SNP dataset, as also confirmed by later KASP assay. To assign SNPs to individual genotypes, KASP markers were designed for validated polymorphic sites and genotyped across all accessions. Genotype-specific SNP profiles were then reconstructed from the KASP data. For each homeolog (*TdBCAT-A* and *TdBCAT-B*), haplotypes were defined as unique combinations of validated SNP alleles across the target sequence. Genotypes sharing identical SNP profiles were assigned to the same haplotype. Combined haplotypes were subsequently generated by pairing the *TdBCAT-A* and *TdBCAT-B* haplotypes present within each genotype. A haplotype combination therefore corresponds to the specific pairing of one *TdBCAT-A* haplotype and one *TdBCAT-B* haplotype present within a genotype.

### SNP assay through Kompetitive Allele Specific PCR

On the variant of *TdBCAT*, KASP markers were designed using the reference genome of *T. durum* cv. Kronos (https://grassroots.tools/service/blast-blastn). All primers were purchased from Sigma-Aldrich (Gillingham, UK); a full list is provided in **Supplementary Table 3**. KASP endpoint genotyping analysis was conducted on Real Time PCR 7900HT (Life Technologies) following the manufacturer’s instruction (LGC Genomics, Teddington, UK). Briefly, KASP assay mix was prepared as follows: 46 μL of dH_2_O, 30 μL of 100 μM common primer, and 12 μL of each 100 μM allele-specific primer. Reactions were set up in 10 μL of final volume including 5 μL of KASP Master mix, 0.14 μL of KASP assay mix, and 10–20 ng of DNA in duplicates. All assays included the amplification of reference DNA (cv. Kronos) as positive control (allele 1). PCR cycling was performed following two protocols depending on the KASP assay: (1) 1 cycle at 94 °C for 15 mins, followed by 10 touchdown cycles (94 °C for 20 s; touchdown 65 °C, -0.8 °C per cycle, 60 s) then followed by 30 cycles of amplification (94 °C 20 s; 57 °C 60 s); and (2) 1 cycle at 94 °C for 15 min, followed by 10 touchdown cycles (94 °C for 20 s; touchdown 61 °C, −0.6 °C per cycle, 60 s) then followed by 30 cycles of amplification (94 °C 20 s; 55 °C 60 s).

### Imaging phenotyping measurements

The HTP experiment was performed in the PhenItaly ALSIA Metaponto Agrobios phenotyping facility (Metaponto, Italy) by acquiring images in the RGB domain (white light) every 2 days as previously indicated by Danzi et al. (2019), by using a Scanalyzer 3D system (LemnaTec GmbH, Aachen, Germany).

Because of the lack of seeds for some of the SSD analyzed in NGS, only 32 genotypes from the SSD collection (SSD35, SSD44, SSD64, SSD69, SSD92, SSD99, SSD109, SSD112, SSD116, SSD122, SSD135, SSD171, SSD178, SSD195, SSD244, SSD253, SSD269, SSD278, SSD322, SSD325, SSD335, SSD343, SSD397, SSD409, SSD415, SSD416, SSD441, SSD451, SSD459, SSD487, SSD494, and SSD511) and three varieties (Svevo, Saragolla, and Cappelli) were germinated at room temperature for 4 days on moist filter paper and then transplanted into polystyrene racks that were stored at 4 °C for 2 weeks to allow the synchronization of the growth cycle. Individual plants were then transferred into 2-L pots filled with a 1:1 (v/v) mixture of river sand and peat moss until a total weight of 1.2 kg is reached. Plants were then grown in a glasshouse under natural light conditions, and environmental conditions were monitored every 30 min using a datalogger (Watchdog Model 450, Spectrum Technologies, Inc). Three treated and three control replicates of each accession were randomly distributed in a greenhouse to minimize the possible establishment of microclimatic variation in the greenhouse through spatial distribution. Each pot carries a barcode allowing the automatic reading of plant identifier. Wheat plants were manually kept fully irrigated up to the booting stage [Z45, 104 days after sowing (DAS)], and then DS was imposed for 43 days (Z60, 147 DAS) by maintaining the amount of water in the soil at approximately 50% of field capacity (FC).

Each plant was imaged sequentially, employing different wavelengths in the visible and non-visible spectrum even if for this investigation only the visible wavelength was considered. The range of imaging involving three mutually orthogonal vantage points was used to evaluate morphometric parameters of the plant, such as height, width, and biomass (Petrozza et al., 2014). The digital biovolume (DB) was calculated from the three orthogonal images of the same plant according to the formula indicated in Danzi et al. (2019).

Visible light images were segmented with LemnaTec LemnaGrid (v5) software using the “RGB Calculator” function with the formula “2*G/(R+B)”, where G, R, and B represent the green, red, and blue values, respectively, for individual pixels. Background removal the Fill areas function with a value of 25 pixels thus filling foreground and background objects of 25 or less pixels. Feature extraction was carried out with the Histogram and then the “Color Class Objects Writer” function for NIR and RGB color values and the “Objects Writer” function for morphometric parameters such as area, compactness, height, and width. The DB was calculated according to Eberius Lima-Guerra, being the area from both side views + log(area from top view)/3.

The digital biovolume ratio (DBR) was expressed as (biovolume of treated plants/biovolume of control plants). Genotype ranking under DS was based on the observed phenotypic index DBR averaged across biological replicates and evaluated at each sampling time point, rather than on BLUP-based estimates across time. Based on the mean DBR values across all DS conditions, genotypes were arbitrarily classified into three resilience categories: highly resilient (DBR ≥ 0.85), moderately resilient (0.80 ≤ DBR < 0.85), and low resilient (DBR < 0.80).

Differences between control and drought-stressed plants were evaluated at each time point using a two-way analysis of variance (ANOVA) with genotype and treatment as fixed factors.

## Results

### Identification of natural variation in *TdBCAT* genes

Deep sequencing of four pools generated corresponding datasets, with mapped read counts ranging from 964,745 to 1,746,314 depending on the target gene (*TdBCAT-A* or *TdBCAT-B*) and pool (1–4) (**Supplementary Table 4**). A total of 93 polymorphic sites were identified, 62 in *TdBCAT-A* and 31 in *TdBCAT-B*, with allele frequencies ranging from 1% to 82%. Each dataset resulted from the NGS of target sequences from 9 (in pools 2 and 4) and 10 genotypes (in pools 1 and 3). Variants with a frequency of less than 5% were considered false positives possibly due to PCR artifacts and data shifts during deep NGS reading.

A total of 27 SNPs with a frequency ≥5% were identified, 20 in *TdBCAT-A* and 7 in *TdBCAT-B*. These SNPs were distributed as follows: 16 in the promoter, 2 in the intron, and 2 in the UTR-3 region of *TdBCAT-A*; and 4 in the promoter, 1 in the UTR-5, 1 in the UTR-3, and 1 in the CDS of *TdBCAT-B*, which led to a missense variant in the protein sequence (A367T). Out of the 21 SNPs detected in regulatory regions with a frequency ≥5%, 20 KASP assays were successfully designed to confirm SNP detection and assign each mutation to its specific genotype.

Following KASP validation, SNP frequencies in individual genotypes ranged from 5% (SNP A24) to 16% (SNP B2 and B3) (**Supplementary Table 5**). A total of 10 haplotypes were identified—5 for *TdBCAT-A* (haplotypes 1-5) and 5 for *TdBCAT-B* (haplotypes 6–10)—as shown in **Figure 2**. Notably, 10 of the 21 SNPs in *TdBCAT-A* belonged to the same haplotype, shared by SSD 409, SSD 441, and SSD 397. For *TdBCAT-B*, the most variable haplotype, with three SNPs, was found in SSD 322. Among the control cultivars, Svevo, Colosseo, and Saragolla shared the same haplotype as the reference *Kronos* for *TdBCAT-A*, while Cappelli displayed the highest variability with three SNPs (**Figure 2**). In contrast, for *TdBCAT-B*, only Saragolla was identical to Kronos, while Cappelli, Colosseo, and Svevo each presented one SNP (**Table 1**, **Figure 2**).

**Figure 2:**
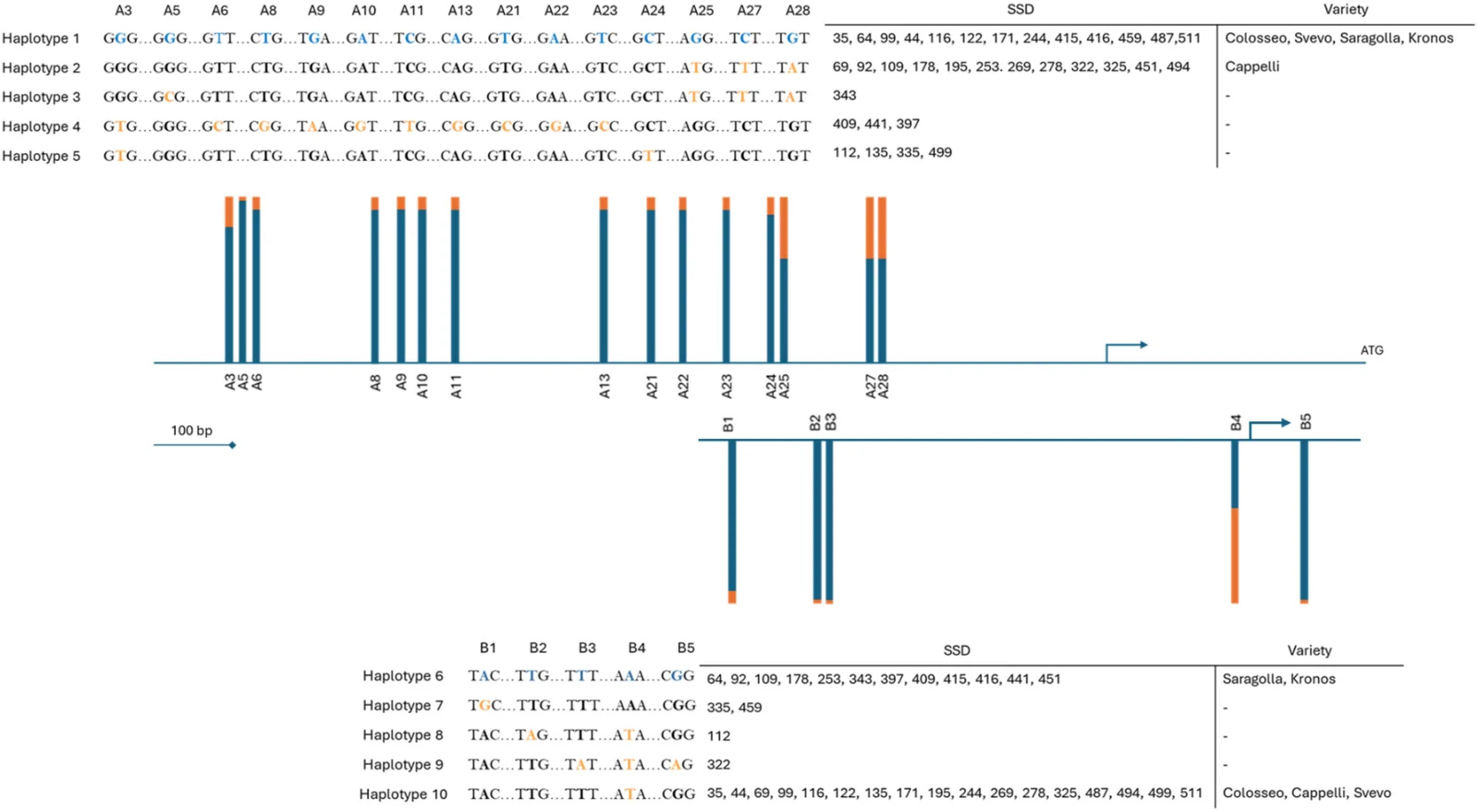
Haplotypes identified in *TdBCAT* promoters of 38 durum wheat genotypes. Haplotype 1 and Haplotype 6 are reference for *TdBCAT-A* and *TdBCAT-B*, respectively.

**Table 1:** List of haplotype combination of *TdBCAT-A* and *-B* genes in the analyzed genotypes.

Haplotype combination	*TdBCAT-A haplotype*	*TdBCAT-B haplotype*	*SNPs*	Genotypes
1	1	6	None	64, 415, 416, Saragolla, Kronos
2	2	6	A25, A27, A28	92, 109, 178, 451
3	3	6	A5, A25, A27, A28	343
4	4	6	A3, A6, A8, A9, A10, A11, A13, A21, A22, A23	397, 409, 441
5	1	7	B1	459
6	2	7	A25, A27, A28, B1	253
7	5	7	A3, A24, B1	335
8	5	8	A3, A24, B2, B4	112
9	2	9	A25, A27, A28, B3, B4, B5	322
10	1	10	B4	35, 44, 99, 116, 122, 171, 244, 487, 511, Colosseo, Svevo
11	2	10	A25, A27, A28, B4	69, 195, 269, 278, 325, 494, Cappelli
12	5	10	A3, A24, B4	135, 499

Combining the five *TdBCAT-A* haplotypes and the five *TdBCAT-B* haplotypes identified across the panel resulted in 12 distinct *TdBCAT* haplotype combinations, representing the specific pairing of one *TdBCAT-A* haplotype and one *TdBCAT-B* haplotype within an individual genotype. Six of these combinations were observed in a single SSD genotype and were therefore considered unique (**Table 1**, **Figure 2**). The results confirmed high genetic diversity within the SSDs, which are representative of a broad germplasm collection (Pignone et al., 2015). Among cultivars, Cappelli stood out as the most genetically diverse, while Saragolla was the closest match to Kronos, with no SNP variation in *TdBCAT-A* or *TdBCAT-B.*

### Variations in the *TdBCAT* genes can affect the phenotype of durum wheat genetic resources in drought stress conditions

RGB imaging has emerged as a reliable, non-invasive method for quantifying morphometric traits in plants, with particular efficacy in detecting drought-induced stress through DB estimation (**Supplementary Table 6**) and the DBR calculated for all genotypes. DBR proved to be a reliable proxy for drought resilience (Janni et al., 2019; Danzi et al., 2022). It reflects a genotype’s growth performance and health under stress conditions as drought, which can negatively impact biomass (Poiré et al., 2014). **Figure 3** illustrates the DBR change over 43 days of monitoring during DS.

**Figure 3:**
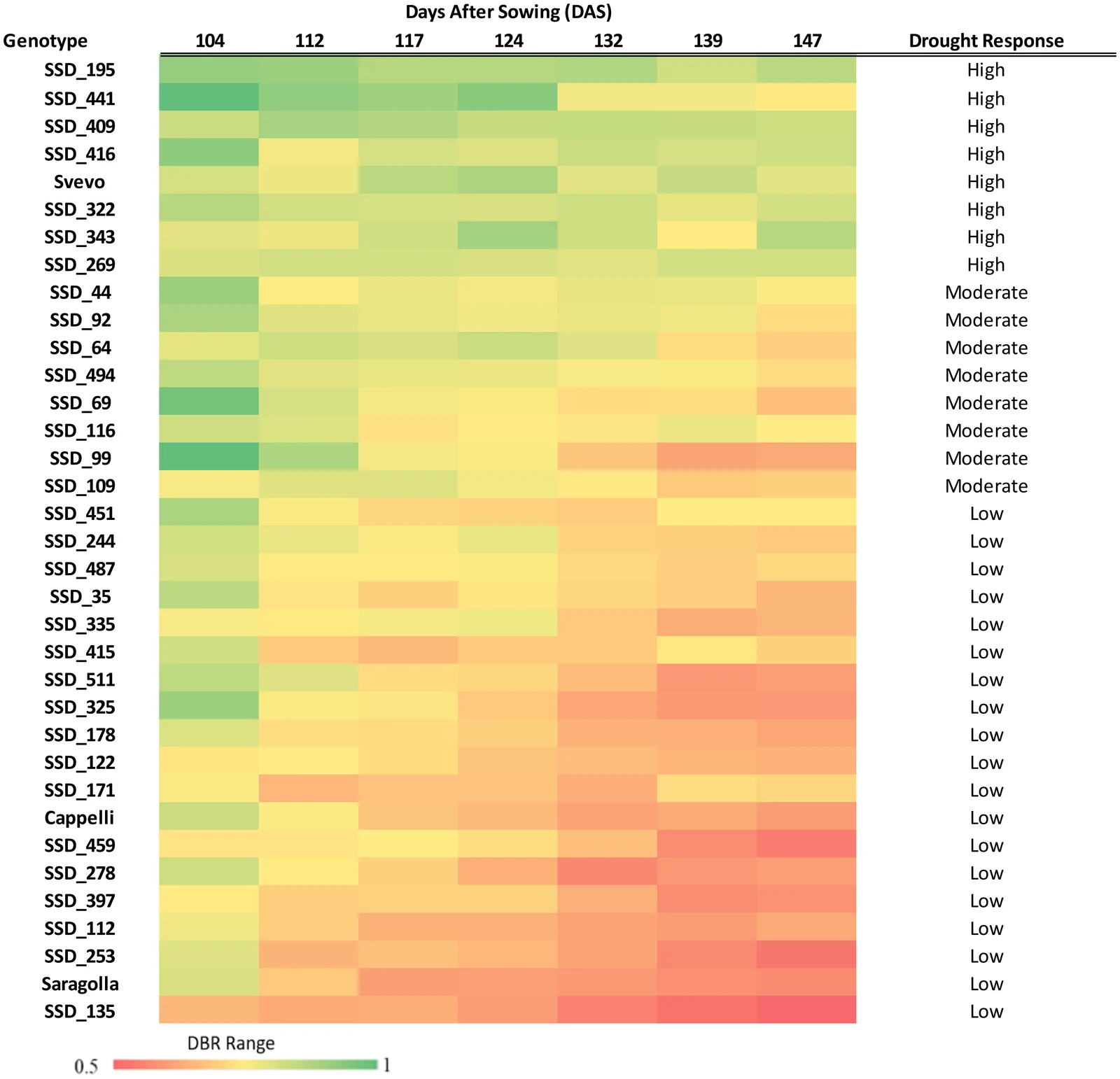
Heat map of the digital biovolume ratio (DBR) for 35 durum wheat genotypes at six different time points. The DB was acquired in the high-throughput phenotyping platform from 104 days after sowing (DAS) when the drought stress was imposed, until 139 DAS. For each genotype, the corresponding classification of low, moderate, and high drought resilience is reported.

A higher DBR indicates greater biomass under stress, reflecting better drought adaptability. In many landraces, a decline in DBR was observed starting at 112 DAS, which persisted throughout the experiment. These results are consistent with similar findings in tomato (Petrozza et al., 2014). DBR reached its lowest point between 132 and 147 DAS, when DS and natural senescence overlapped (**Figure 3**; **Supplementary Table 6**), with the sole exception of three genotypes at 112 DAS (SSD 44, SSD 416, and Svevo).

Based on the mean DBR from 104 to 147 DAS, genotypes were categorized into three major groups characterized by high, moderate, and low drought resilience. Eight genotypes (SSD 416, SSD 195, SSD 343, SSD 409, SSD 322, SSD 269, SSD 441, and Svevo) demonstrated high resilience (DBR ≥ 0.85), with SSD 441, SSD 409, and SSD 195 showing the most stable DBR (mean > 0.9). Eight genotypes (SSD 44, SSD 92, SSD 64, SSD 494, SSD 69, SSD 116, SSD 99, and SSD 109) were classified as moderately resilient (0.85 < DBR ≤ 0.8). Genotypes with DBR < 0.8 were considered with low resilience (SSD 451, SSD 244, SSD 487, SSD 35, SSD 335, SSD 415, SSD 511, SSD 325, SSD 178, SSD 122, SSD 171, Cappelli, SSD 459, SSD 278, SSD 397, SSD 112, SSD 253, Saragolla, and SSD 135).

In summary, Svevo showed the greatest stability under DS based on DBR (**Table 2**), while Cappelli did not confirm its reported adaptability under the experimental conditions used in this study.

**Table 2:** Summary of drought resilience classification (high–moderate–low) based on the average digital biovolume ratio (DBR) between 104 and 147 days after sowing (DAS).

Degree of tolerance	Haplotype													Geographic region
	*TdBCAT-A*	1	2	3	4	1	2	5	5	2	1	2	5	
	*TdBCAT-B*	6	6	6	6	7	7	7	8	9	10	10	10	
	Haplotype combination	1	2	3	4	5	6	7	8	9	10	11	12	
	DBR													
High												195		Saudi Arabia
High					441									Crete
High					409									Greece
High		416												Greece
High											Svevo			Italy
High										322				Turkey
High				343										Iran
High												269		Iran
Moderate											44			Tunisia
Moderate			92											USA—North Dakota
Moderate		64												Morocco
Moderate												494		Greece
Moderate												69		Morocco
Moderate											116			Iraq
Moderate											99			Ethiopia
Moderate			109											Iraq
Low			451											Iraq
Low											244			Ethiopia
Low											487			Greece
Low											35			Algeria
Low								335						Iraq
Low		415												Crete
Low											511			Libya
Low												325		Syria
Low			178											France
Low											122			USA—North Dakota
Low											171			Perù
Low												Cappelli		Italy
Low						459								USA
Low												278		Bulgaria
Low					397									Crete
Low									112					Iraq
Low							253							Cyprus
Low		Saragolla												Italy
Low													135	Turkey

Green boxes represent high drought resilience (average DBR > 0.85), yellow boxes denote moderate drought resilience (average DBR 0.80 < *x* < 0.85), and orange boxes indicate low drought resilience (average DBR < 0.8). For each genotype (SSD or cultivars), the list of haplotype combination of *TdBCAT* assessed by the allele mining approach is presented along with the geographic origin.

SSD 441, SSD 409, and SSD 397 carried the most polymorphic *TdBCAT-A* haplotype (haplotype 4; **Table 1**, **Figure 2**). Although SSD 441 and SSD 409 exhibited high resilience based on DBR, SSD 397 showed low resilience (**Table 2**). In addition, SSD 459, SSD 253, SSD 112, SSD 135, and SSD 322 carried unique haplotype combination and were generally classified as low-resilient (**Table 2**). Notably, haplotype 7 of *TdBCAT-B* was only present in low-resilient genotypes.

## Discussion

Since its domestication more than 10,000 years ago, wheat has undergone continuous selection that has substantially increased productivity and adaptation to diverse agro-climatic conditions. However, this remarkable breeding success has also narrowed the genetic diversity available within elite germplasm (Lopes et al., 2015; Pignone et al., 2016). This reduced genetic diversity limits the development of improved wheat varieties required to sustainably meet the food demands of a growing global population, particularly in the face of climate change and increasing abiotic and biotic stresses (Sansaloni et al., 2020). Consequently, exploiting the untapped allelic diversity preserved in landraces has become a major priority for modern breeding.

Recent advances in genomics have revolutionized the exploration of natural genetic variation. Genotyping-by-sequencing (GBS) enables the identification of thousands of SNPs, providing dense molecular markers for genome mapping in wheat and other cereals (Hussain et al., 2017; Thabet et al., 2018). Nevertheless, despite the power of NGS, its routine application to large breeding populations remains economically challenging. In this context, our study demonstrates that combining NGS-based SNP discovery with KASP genotyping provides a cost-effective and efficient strategy for allele mining in durum wheat, accelerating the identification of favorable alleles for breeding (Yang and Qin, 2023).

A complex challenge lies in linking genetic variation to complex adaptive traits. While genomic resources have expanded rapidly, large-scale phenotyping remains a major bottleneck in dissecting quantitative traits (Singh et al., 2019; Morisse et al., 2022). Identifying genetic variants in single genes that contribute to phenotypic variation under selective pressure holds great potential for improving genetic breeding, natural resource conservation, and crop management (Cullingham et al., 2020; Khadka et al., 2020; Guadarrama-Escobar et al., 2024).

Gene expression analyses support the role of *TdBCAT* genes in drought resilience (Buffagni et al., 2020). In this study, two haplotype combinations of *TdBCAT* genes, 10 and 11, showed the highest frequency among SSD genotypes (nine and six SSDs, respectively; **Table 1**). However, the high-performing haplotype combinations under drought conditions were 1, 2, 3, 4, 9, 10 and 11 (**Table 2**). Based on the geographic origins of the SSDs and the variation in *TdBCAT* alleles, we created a gene ecology map for the analyzed SSDs (**Figure 4**). Some haplotype combinations, such as 1, 4, and 6, are characteristic of Mediterranean genotypes, while others, including 2, 3, 7, 8, 9, 11, and 12, are mostly found in the Fertile Crescent, highlighting a higher genetic diversity in those regions (Janni et al., 2018).

**Figure 4:**
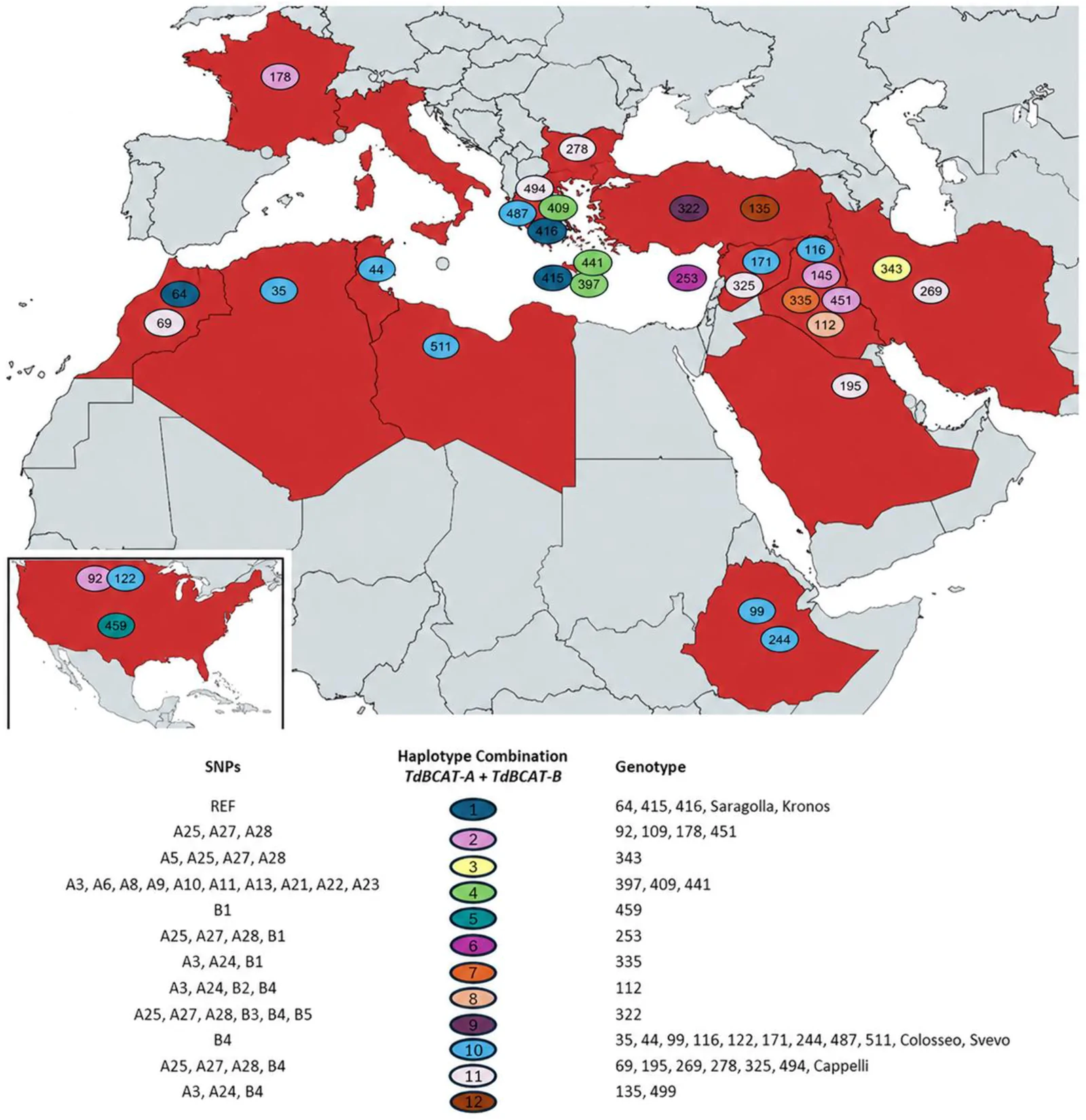
Distribution of SSDs evaluated for their drought tolerance with HTTP. SSDs are positioned on the map according to their country of origin. Each SSD is presented with an oval, which is differently colored according to the haplotype combination of *TdBCAT-A* and *TdBCAT-B* promoters.

Genotypes originating from the Mediterranean (Greece and Crete) and the Fertile Crescent (Iran, Iraq, and Turkey) showed greater drought tolerance, linked to *TdBCAT* allele variation. Haplotypes 3 and 9, which are unique to one genotype each, further suggest that new superior alleles can be found in genotypes from these regions (Janni et al., 2018).

This aligns with previous studies showing that Mediterranean durum wheat landraces represent a vast genetic diversity and specific adaptations to local environments (Nazco et al., 2012).

Haplotype combinations 1, 10, and 11 are present in several genotypes from broad geographic origins and exhibit highly variable phenotypes under DS, suggesting that they do not play a key role in drought resistance. However, the high conservation of their CDSs indicates that evolutionary adaptation was more likely mediated by variation in regulatory elements, which may have facilitated adaptation to diverse and challenging agricultural environments.

This study also provided insights into the drought response of three widely cultivated wheat varieties: Svevo, Cappelli, and Saragolla. Svevo demonstrated strong drought resilience by maintaining a high DB and tissue water content during DS, while Saragolla showed poor resilience.

Cappelli, under the experimental conditions used in this study, did not confirm its reported adaptability to drought, showing a low DB, similar to control plants.

Combining novel genomic approach and high-throughput phenotyping analysis, new potential genetic sources of drought resilience were identified despite a significant variability, within the landrace genomes. Out of the 32 SSDs, 3 to 6 were identified as promising candidates for wheat breeding programs based on phenotypic analyses.

At this stage, it is challenging to directly match haplotypes to specific phenotypes due to the genetic variability in landrace genomes. However, our results suggest that *TdBCAT* variants play a role in regulating DS responses, as supported by previous phenotypic studies on the same SSD population (Danzi et al., 2022).

In conclusion, the proposed cost-effective SNP mining approach enabled the identification of different haplotype combinations within the complex genetic background of durum wheat landraces, highlighting its effectiveness for characterizing durum wheat germplasm collections. When coupled with an HTP approach, it facilitated the identification of genotypes with greater adaptability to drought conditions, demonstrating the value of HTP for sustainable variety selection. Nevertheless, further phenotypic analyses are required to validate these findings and better characterize their adaptive responses. These results can be used for pre-breeding selection, particularly when starting from durum wheat landraces, which provide a valuable genetic reservoir of allelic variation in traits of interest (Lopes et al., 2015; Janni et al., 2018; Branlard et al., 2020).

## Conclusions

- The combination of targeted SNP mining and HTP efficiently identifies drought-tolerant genotypes from a core set of durum wheat germplasm.
- *TdBCAT-A* and *TdBCAT-B* genes exhibit high genome stability within their CDSs, while the greatest SNP variability is found in their promoter regions.
- A clear link between identified haplotypes and their geographic origins highlights the significant genetic diversity present in the germplasm collections.
- DB was confirmed as a reliable indicator of durum wheat’s drought resilience at the phenotypic level.

## Data Availability

The original contributions presented in the study are publicly available. This data can be found here: https://www.ncbi.nlm.nih.gov/bioproject/PRJNA1521933.
