# Supplementary material for: Uncovering natural variation in TdBCAT genes and phenotypic variation under drought in durum wheat
Source: Front Plant Sci. 2026 Sep 14;17:1826616. doi: 10.3389/fpls.2026.1826616 (PMC13617607; doi:10.3389/fpls.2026.1826616)
Supplement: Supplementary file 1 [file SupplementaryFile2.docx]

**Supplementary Figures**

**Supplementary Figure 1.** Gene models of A) *TdBCAT-A* and B) *TdBCAT-B* and primer pairs (PPs) designed for the target enrichment by PCR. The black boxes correspond to exons (1-7). Each targeted region amplified by PCR is shown with a continuous line and arrows, which represent primer pairs used to amplify target regions.

**Supplementary Tables**

**Suppl. Table 1.** List of  SSD and Passport information. The MGD Accession Number can be used to search in the MGD database to collect passport information (<https://www.ibbr.cnr.it/mgd/?action=search&p=str>).

| **SSD #** | **MGD Accession Number** | **Country of origin** |
| --- | --- | --- |
| 35 | 70 | ALGERIA |
| 44 | 96 | TUNISIA |
| 64 | 345 | MAROCCO |
| 69 | 351 | MAROCCO |
| 92 | 423 | USA - ND NORTH DAKOTA |
| 99 | 477 | ETIOPIA |
| 109 | 496 | IRAQ |
| 112 | 500 | IRAQ |
| 116 | 505 | IRAQ |
| 122 | 578 | USA - ND NORTH DAKOTA |
| 135 | 618 | TURCHIA |
| 171 | 1105 | PERU' |
| 178 | 1199 | FRANCIA |
| 195 | 1384 | ARABIA SAUDITA |
| 244 | 1830 | ETIOPIA |
| 253 | 1894 | CIPRO |
| 269 | 1984 | IRAN |
| 278 | 2070 | BULGARIA |
| 322 | 2561 | TURCHIA |
| 325 | 2675 | SIRIA |
| 335 | 2961 | IRAQ |
| 343 | 2977 | IRAN |
| 397 | 17667 | CRETA |
| 409 | 17691 | GRECIA |
| 415 | 17711 | CRETA |
| 416 | 17712 | GRECIA |
| 441 | 17841 | CRETA |
| 451 | 27815 | IRAQ |
| 459 | 28674 | USA |
| 487 | 5099 | GRECIA |
| 494 | 5572 | GRECIA |
| 511 | 7388 | LIBIA |

| **Supplementary Table 2**. List of primer pairs (PPs) used for the targeting of *TdBCAT* gene sequences by PCR before NGS, along with 5’-3’ sequence, annealing temperature and amplicon size. | | | | | | |
| --- | --- | --- | --- | --- | --- | --- |
| **Target gene** | **Primer pair** | **Upstream primer** | **Downstream primer** | **T annealing (°C)** | **Product Size (bp)** |  |
|  |  | **(5'-3' sequence)** | **(5'-3' sequence)** |  |  |  |
| ***TdBCAT-A*** | ***PP-1*** | GGTAGTGGCCGACGAAGG | CAGACGACAGCACAGCCAT | 56 *(a)* | 1661 |  |
| ***TdBCAT-B*** | ***PP-2*** | GCCTTGACACGACGACTTTC | CAGACGACAGCACAGCCAT | 56 *(a)* | 975 |  |
| ***TdBCAT-A*** | ***PP-3*** | CTCCACGACCTATATAGAAACCTC | GAGCTCGATGATGCTCCGA | 56 | 1722 |  |
| ***TdBCAT-B*** | ***PP-4*** | CTCCACGACCTATATAGAAACCTC | GAGCTCGATGATGCTCCTG | 56 | 1760 |  |
| ***TdBCAT-A*** | ***PP-5*** | GAGATCCACCGCGCCATG | CAGTAGCATCAGAGAAACCATC | 60 *(a)* | 945 |  |
| ***TdBCAT-B*** |  |  |  | 60 *(a)* | 925 |  |
| *(a) final annealing temperature (x35 cycles) of Touch-down PCR* | | | | | | |

**Supplementary Table 3.** Full list of KASP assay used for the SNP assignment. Each KASP assay is composed of three primers: a common primer and two allele-specific primers carrying at the 5’ the FAM or VIC/HEX complementary sequence for the reference (Allele 1) and alternative allele (Allele 2) respectively. (FAM tail: 5' GAAGGTGACCAAGTTCATGCT 3', HEX tail: 5' GAAGGTCGGAGTCAACGGATT 3').

| **Target** | **SNP** | **Common** | **Allele 1** | **Allele 2** | **Assay** |
| --- | --- | --- | --- | --- | --- |
| *TdBCAT-A* | A3 | TGGCAAATCCCCATCCCTC | (VIC/HEX)-ACAAAGGGCTCTGATGGGG | (FAM)-GACAAAGGGCTCTGATGGGT | 1 |
| *TdBCAT-A* | A5 | CGACAAAGGGCTCTGATGGG | (VIC/HEX)-TTCCCATCGAAACGCGCC | (FAM)-TTCCCATCGAAACGCGCG | 1 |
| *TdBCAT-A* | A6 | CGACAAAGGGCTCTGATGGG | (VIC/HEX)-TCCCTCGTTTCCCATCGAAA | (FAM)-TCCCTCGTTTCCCATCGAAG | 1 |
| *TdBCAT-A* | A8 | AGCACAACCTCTTAGTCCCC | (VIC/HEX)-GGCGACGCCATTGACT | (FAM)-GGCGACGCCATTGACG | 1 |
| *TdBCAT-A* | A9 | TTTTCTAGTGGTATATCGGTGTGA | (VIC/HEX)-GCTAAAACTTCCCTACGCCATTG | (FAM)-GCTAAAACTTCCCTACGCCATTA | 1 |
| *TdBCAT-A* | A10 | TTTTCTAGTGGTATATCGGTGTGA | (VIC/HEX)-GACGGGGCTGCGTGGA | (FAM)-GACGGGGCTGCGTGGG | 1 |
| *TdBCAT-A* | A11 | TTTTCTAGTGGTATATCGGTGTGA | (VIC/HEX)-ACAGTTCCTACCAAAATTACCATATC | (FAM)-ACAGTTCCTACCAAAATTACCATATT | 1 |
| *TdBCAT-A* | A13 | ACACCATCCGACCATGCTAG | (VIC/HEX)-GAAGGTGAGACGACGGCA | (FAM)-AAGGTGAGACGACGGCG | 1 |
| *TdBCAT-A* | A21 | GTCTCGGTGGTGCGTCTA | (VIC/HEX)-CCAGAGACAGACATCCACACA | (FAM)-CAGAGACAGACATCCACACG | 1 |
| *TdBCAT-A* | A22 | GTCTCGGTGGTGCGTCTA | (VIC/HEX)-AGACAATCCCCGACCGAATT | (FAM)-AGACAATCCCCGACCGAATC | 1 |
| *TdBCAT-A* | A23 | GTCTCGGTGGTGCGTCTA | (VIC/HEX)-GCCGCATGAACGTAGATGA | (FAM)-GCCGCATGAACGTAGATGG | 1 |
| *TdBCAT-A* | A24 | CACCGTCCCTCCCTTGTC | (VIC/HEX)-TCTTCATCAGCGGTGTTTGC | (FAM)-CTCTTCATCAGCGGTGTTTGT | 1 |
| *TdBCAT-A* | A25 | CACCGTCCCTCCCTTGTC | (VIC/HEX)-CTGGTGCGCTGGTCCTATAG | (FAM)-CTGGTGCGCTGGTCCTATAT | 1 |
| *TdBCAT-A* | A27 | TCCCGAAAATACTTCCATTCTGT | (VIC/HEX)-CCTTCGGCTCACTTCAATTC | (FAM)-CCTTCGGCTCACTTCAATTT | 1 |
| *TdBCAT-A* | A28 | GGAGGGACGGTGACAGT | (VIC/HEX)-ATAATAAAAGATACAGTCAAATCCAC | (FAM)-ATAATAAAAGATACAGTCAAATCCAT | 1 |
| *TdBCAT-B* | B1 | GTTGGAGCTGGGCATAACTTA | (VIC/HEX)-ACGACGACTTTCCGACTATCTA | (FAM)-CGACGACTTTCCGACTATCTG | 2 |
| *TdBCAT-B* | B2 | GCCCAGCTAATTTTTTCCAA | (VIC/HEX)-ATTTTTTATTATTTTAGGTGTTCATT | (FAM)-ATTTTTTATTATTTTAGGTGTTCATA | 2 |
| *TdBCAT-B* | B3 | GCCCAGCTAATTTTTTCCAA | (VIC/HEX)-GGTGTTCATTGTACTGTTATAATATT | (FAM)-GGTGTTCATTGTACTGTTATAATATA | 2 |
| *TdBCAT-B* | B4 | CAGCGAGTTAAAATGGCAACC | (VIC/HEX)-GCCAAATTCTCCACGACCTAA | (FAM)-GCCAAATTCTCCACGACCTAT | 1 |
| *TdBCAT-B* | B5 | CGAGTACCTAGCGGCTCC | (VIC/HEX)-CGAGCAATATAATGGCCAGCC | (FAM)-CGAGCAATATAATGGCCAGCT | 1 |

**Supplementary Table 4**. Summary of the number of mapped reads from NGS on the 4 pool of TdBCAT amplified by targeted PCR.

|  |  |  |  | **Number of mapped reads** | | | |
| --- | --- | --- | --- | --- | --- | --- | --- |
| **Gene** | **Kronos Scaffold** | **Position** | **Strand** | **POOL 1** | **POOL 2** | **POOL 3** | **POOL 4** |
| TdBCAT-A | Triticum_turgidum_Kronos_Elv1.1_scaffold_059708 | 14825-11061 | minus | 1,385,670 | 964,745 | 1,070,464 | 1,254,931 |
| TdBCAT-B | Triticum_turgidum_Kronos_Elv1.1_scaffold_014525 | 8379-95360 | minus | 1,746,314 | 1,320,628 | 1,584,427 | 1,409,427 |

**Supplementary Table 5**. List of SNPs discovered in *TdBCAT* promoters. For each variant the nucleotide change is indicated with the position on the isolated 5’-3’ sequence of the relative gene. Variants frequency (%) at SNP are presented for each pool, when detected.

|  |  |  | **Variant Frequency** | | | |
| --- | --- | --- | --- | --- | --- | --- |
| **Gene** | **SNP** | **Nucleotide change** | **Pool 1** | **Pool 2** | **Pool 3** | **Pool 4** |
| *TdBCAT-A* | A3 | G100T | - | 9% | 9% | - |
| *TdBCAT-A* | A5 | G118C | - | 11% | - | - |
| *TdBCAT-A* | A6 | T124C | - | 10% | 10% | - |
| *TdBCAT-A* | A7 | A139G | - | - | 7% | - |
| *TdBCAT-A* | A8 | T283G | - | 10% | 10% | - |
| *TdBCAT-A* | A9 | G333A | - | 10% | 10% | - |
| *TdBCAT-A* | A10 | A348G | - | 10% | 10% | - |
| *TdBCAT-A* | A11 | C399T | - | 10% | 10% | - |
| *TdBCAT-A* | A13 | A592G | - | 10% | 10% | - |
| *TdBCAT-A* | A21 | T659C | - | 10% | 10% | - |
| *TdBCAT-A* | A22 | A692G | - | 10% | 10% | - |
| *TdBCAT-A* | A23 | T742C | - | 10% | 9% | - |
| *TdBCAT-A* | A24 | C810T | - | - | 5% | 37% |
| *TdBCAT-A* | A25 | G834T | 53% | 48% | 35% | 38% |
| *TdBCAT-A* | A27 | C938T | 53% | 48% | 38% | 35% |
| *TdBCAT-A* | A28 | G962A | 53% | 48% | 39% | 35% |
| *TdBCAT-B* | B1 | A30G | - | - | 7% | 10% |
| *TdBCAT-B* | B2 | T163A | - | - | - | 16% |
| *TdBCAT-B* | B3 | T179A | - | 16% | - | - |
| *TdBCAT-B* | B4 | A690T | 75% | 79% | 81% | 82% |
| *TdBCAT-B* | B5 | G797A | - | 11% | - | - |

**Suppl. Table 6**. Digital Biovolume Ratio measured with high-thoughtput phenotyping platform (HTTP) throughout the entire experiment.

| **DAS** | | | | | | | | | | | | | | | | | | |
| --- | --- | --- | --- | --- | --- | --- | --- | --- | --- | --- | --- | --- | --- | --- | --- | --- | --- | --- |
| **SSD** | **55** | **62** | | **76** | **92** | | **104** | **112** | | **117** | **124** | | **132** | **139** | | **147** | **167** |  |
| **SSD_35** | 1.233 | 1.125 | | 0.985 | 1.038 | | 0.929 | 0.778 | | 0.740 | 0.779 | | 0.750 | 0.733 | | 0.685 | 0.803 |  |
| **SSD_44** | 1.032 | 0.925 | | 0.934 | 1.115 | | 0.988 | 0.802 | | 0.837 | 0.819 | | 0.843 | 0.842 | | 0.805 | 0.897 |  |
| **SSD_64** | 1.058 | 0.969 | | 0.950 | 1.011 | | 0.849 | 0.895 | | 0.871 | 0.902 | | 0.861 | 0.765 | | 0.734 | 0.867 |  |
| **SSD_69** | 0.975 | 1.099 | | 1.229 | 1.089 | | 1.073 | 0.881 | | 0.819 | 0.809 | | 0.760 | 0.769 | | 0.703 | 0.877 |  |
| **SSD_92** | 0.928 | 0.941 | | 1.071 | 1.028 | | 0.960 | 0.858 | | 0.842 | 0.821 | | 0.838 | 0.827 | | 0.760 | 0.929 |  |
| **SSD_99** | 1.109 | 0.962 | | 0.946 | 1.190 | | 1.104 | 0.958 | | 0.815 | 0.807 | | 0.718 | 0.650 | | 0.659 | 0.902 |  |
| **SSD_109** | 1.026 | 1.077 | | 1.069 | 0.997 | | 0.812 | 0.857 | | 0.866 | 0.819 | | 0.787 | 0.728 | | 0.739 | 0.944 |  |
| **SSD_112** | 1.162 | 0.990 | | 0.986 | 0.999 | | 0.825 | 0.734 | | 0.676 | 0.676 | | 0.644 | 0.633 | | 0.661 | 0.756 |  |
| **SSD_116** | 0.916 | 1.009 | | 0.974 | 0.984 | | 0.892 | 0.867 | | 0.772 | 0.794 | | 0.782 | 0.833 | | 0.796 | 0.865 |  |
| **SSD_122** | 1.040 | 0.849 | | 0.815 | 0.916 | | 0.778 | 0.798 | | 0.761 | 0.713 | | 0.699 | 0.683 | | 0.676 | 0.824 |  |
| **SSD_135** | 0.935 | 0.845 | | 0.792 | 0.654 | | 0.687 | 0.663 | | 0.668 | 0.631 | | 0.577 | 0.547 | | 0.527 | 0.616 |  |
| **SSD_171** | 0.935 | 0.947 | | 0.869 | 0.919 | | 0.810 | 0.689 | | 0.712 | 0.712 | | 0.671 | 0.765 | | 0.749 | 0.786 |  |
| **SSD_178** | 1.026 | 0.758 | | 0.832 | 0.820 | | 0.866 | 0.766 | | 0.763 | 0.739 | | 0.676 | 0.673 | | 0.653 | 0.859 |  |
| **SSD_195** | 1.131 | 1.194 | | 1.079 | 1.281 | | 1.005 | 0.991 | | 0.942 | 0.944 | | 0.952 | 0.888 | | 0.932 | 1.038 |  |
| **SSD_244** | 1.041 | 0.965 | | 0.813 | 1.042 | | 0.883 | 0.843 | | 0.806 | 0.842 | | 0.745 | 0.740 | | 0.724 | 0.796 |  |
| **SSD_253** | 0.923 | 0.995 | | 0.957 | 0.907 | | 0.861 | 0.681 | | 0.707 | 0.687 | | 0.650 | 0.592 | | 0.556 | 0.649 |  |
| **SSD_269** | 0.983 | 0.840 | | 0.778 | 0.729 | | 0.869 | 0.886 | | 0.885 | 0.872 | | 0.853 | 0.883 | | 0.885 | 0.900 |  |
| **SSD_278** | 0.989 | 1.101 | | 1.181 | 0.913 | | 0.892 | 0.794 | | 0.741 | 0.673 | | 0.590 | 0.623 | | 0.640 | 0.779 |  |
| **SSD_322** | 1.291 | 1.170 | | 1.045 | 0.995 | | 0.938 | 0.887 | | 0.878 | 0.874 | | 0.894 | 0.843 | | 0.885 | 1.033 |  |
| **SSD_325** | 1.190 | 1.021 | | 0.957 | 0.951 | | 0.987 | 0.804 | | 0.781 | 0.723 | | 0.654 | 0.627 | | 0.625 | 0.718 |  |
| **SSD_335** | 1.089 | 1.116 | | 0.968 | 1.027 | | 0.812 | 0.791 | | 0.816 | 0.827 | | 0.724 | 0.666 | | 0.686 | 0.815 |  |
| **SSD_343** | 0.891 | 0.929 | | 0.913 | 0.779 | | 0.854 | 0.831 | | 0.889 | 0.979 | | 0.897 | 0.796 | | 0.937 | 0.898 |  |
| **SSD_397** | 1.311 | 1.091 | | 1.083 | 0.795 | | 0.792 | 0.737 | | 0.743 | 0.743 | | 0.672 | 0.603 | | 0.615 | 0.674 |  |
| **SSD_409** | 1.011 | 1.048 | | 1.005 | 1.039 | | 0.902 | 0.968 | | 0.948 | 0.907 | | 0.908 | 0.907 | | 0.896 | 1.016 |  |
| **SSD_415** | 0.916 | 0.967 | | 1.035 | 0.947 | | 0.890 | 0.728 | | 0.693 | 0.728 | | 0.726 | 0.783 | | 0.741 | 0.823 |  |
| **SSD_416** | 1.174 | 1.173 | | 1.072 | 1.033 | | 1.017 | 0.820 | | 0.880 | 0.864 | | 0.904 | 0.880 | | 0.893 | 1.049 |  |
| **SSD_441** | 1.172 | 1.109 | | 1.134 | 1.074 | | 1.102 | 1.015 | | 0.979 | 1.030 | | 0.823 | 0.821 | | 0.790 | 1.102 |  |
| **SSD_451** | 1.052 | 1.041 | | 1.189 | 1.106 | | 0.962 | 0.808 | | 0.750 | 0.743 | | 0.732 | 0.795 | | 0.793 | 0.896 |  |
| **SSD_459** | 0.979 | 0.841 | | 0.917 | 0.833 | | 0.777 | 0.778 | | 0.796 | 0.766 | | 0.705 | 0.600 | | 0.564 | 0.725 |  |
| **SSD_487** | 0.860 | 1.013 | | 1.014 | 0.930 | | 0.873 | 0.793 | | 0.791 | 0.809 | | 0.757 | 0.737 | | 0.758 | 0.807 |  |
| **SSD_494** | 1.096 | 1.003 | | 1.066 | 1.031 | | 0.921 | 0.853 | | 0.842 | 0.834 | | 0.811 | 0.808 | | 0.760 | 0.815 |  |
| **SSD_511** | 0.986 | 0.787 | | 0.964 | 0.773 | | 0.925 | 0.860 | | 0.760 | 0.752 | | 0.696 | 0.623 | | 0.639 | 0.755 |  |
| **Cappelli** | 0.807 | 0.919 | | 0.941 | 0.905 | | 0.898 | 0.808 | | 0.717 | 0.690 | | 0.648 | 0.664 | | 0.632 | 0.798 |  |
| **Saragolla** | 0.910 | 0.969 | | 0.984 | 1.083 | | 0.871 | 0.724 | | 0.637 | 0.639 | | 0.627 | 0.607 | | 0.599 | 0.788 |  |
| **Svevo** | 0.759 | 0.843 | | 0.886 | 0.968 | | 0.878 | 0.829 | | 0.935 | 0.961 | | 0.854 | 0.912 | | 0.851 | 0.930 |  |
|  |  |  | |  |  | |  |  | |  |  | |  |  | |  |  |  |
| **ONEWAY ANOVA** | | | | | | | | | | | | | | | | | | |
|  | (F = 0,79; *p* = 0,783) | (F = 1,19; *p* = 0,046) | | (F = 1,89; *p* = 0,01) | (F = 1,15; *p* = 0,301) | | (F = 3,26; *p* = 0,000) | (F = 3,90; *p* = 0,000) | | (F = 3,22; *p* = 0,000) | (F = 3,17; *p* = 0,000) | | (F = 3,31; *p* = 0,000) | (F =4,42; *p* = 0,000) | | (F =4,06; *p* = 0,000) | (F =1,88; *p* = 0,011) |  |
